# Effectiveness of a Web-based Intervention Aimed at Healthy Dietary and Physical Activity Behavior: A Randomized Controlled Trial About Users and Usage

**DOI:** 10.2196/jmir.1624

**Published:** 2011-04-14

**Authors:** Saskia M Kelders, Julia E.W.C Van Gemert-Pijnen, Andrea Werkman, Nicol Nijland, Erwin R Seydel

**Affiliations:** ^3^Netherlands Nutrition CentreDen HaagNetherlands; ^2^National Institute for Public Health and the EnvironmentBilthovenNetherlands; ^1^Department of Psychology, Health and TechnologyFaculty of Behavioural SciencesUniversity of TwenteEnschedeNetherlands

**Keywords:** Randomized controlled trial, usage, eHealth, intervention, attrition, Internet, adherence, retention

## Abstract

**Background:**

Recent studies have shown the potential of Web-based interventions for changing dietary and physical activity (PA) behavior. However, the pathways of these changes are not clear. In addition, nonusage poses a threat to these interventions. Little is known of characteristics of participants that predict usage.

**Objective:**

In this study we investigated the users and effect of the Healthy Weight Assistant (HWA), a Web-based intervention aimed at healthy dietary and PA behavior. We investigated the value of a proposed framework (including social and economic factors, condition-related factors, patient-related factors, reasons for use, and satisfaction) to predict which participants are users and which participants are nonusers. Additionally, we investigated the effectiveness of the HWA on the primary outcomes, self-reported dietary and physical activity behavior.

**Methods:**

Our design was a two-armed randomized controlled trial that compared the HWA with a waiting list control condition. A total of 150 participants were allocated to the waiting list group, and 147 participants were allocated to the intervention group. Online questionnaires were filled out before the intervention period started and after the intervention period of 12 weeks. After the intervention period, respondents in the waiting list group could use the intervention. Objective usage data was obtained from the application itself.

**Results:**

In the intervention group, 64% (81/147) of respondents used the HWA at least once and were categorized as “users.” Of these, 49% (40/81) used the application only once. Increased age and not having a chronic condition increased the odds of having used the HWA (age: beta *=* 0.04, *P* = .02; chronic condition: beta *=* 2.24, *P* = .003). Within the intervention group, users scored better on dietary behavior and on knowledge about healthy behavior than nonusers (self-reported diet: χ^2^_2_ = 8.4, *P* = .02; knowledge: F_1,125_ = 4.194, *P* = .04). Furthermore, users underestimated their behavior more often than nonusers, and nonusers overestimated their behavior more often than users (insight into dietary behavior: χ^2^_2_ = 8.2, *P* = .02). Intention-to-treat analyses showed no meaningful significant effects of the intervention. Exploratory analyses of differences between pretest and posttest scores of users, nonusers, and the control group showed that on dietary behavior only the nonusers significantly improved (effect size *r* = −.23, *P* = .03), while on physical activity behavior only the users significantly improved (effect size *r* = −.17, *P* = .03).

**Conclusions:**

Respondents did not use the application as intended. From the proposed framework, a social and economic factor (age) and a condition-related factor (chronic condition) predicted usage. Moreover, users were healthier and more knowledgeable about healthy behavior than nonusers. We found no apparent effects of the intervention, although exploratory analyses showed that choosing to use or not to use the intervention led to different outcomes. Combined with the differences between groups at baseline, this seems to imply that these groups are truly different and should be treated as separate entities.

**Trial registration:**

Trial ID number: ISRCTN42687923; http://www.controlled-trials.com/ISRCTN42687923/ (Archived by WebCite at http://www.webcitation.org/5xnGmvQ9Y)

## Introduction

The increasing prevalence of overweight is a problem in modern society. It is closely related to a number of chronic conditions such as type 2 diabetes mellitus and places a great burden on the health care system. Losing weight and especially preventing weight regain is challenging. It might be more cost-efficient to prevent people from becoming overweight by focusing on healthy dietary and physical activity (PA) behavior [1-3]. To achieve this goal, interventions aimed at the general public are needed that must not only inform people about the risks of unhealthy dietary and physical activity habits but must also stimulate people to adopt healthier behaviors related to diet and physical activity [2,4]. Previous research has shown that tailored and interactive interventions can achieve this goal [2,4-7]. The Internet provides an opportunity for these interventions to reach a broad population. Besides, by using a Web-based application, the content of the intervention can be tailored to the users, and the intensity can be varied according to the needs and wishes of these users [8-9]. Research has already shown the potential of these applications for the achievement of weight loss and weight management [6,10-14]. However, most studies are focused on applications aimed at treatment or secondary prevention. Many questions remain about the users and the effectiveness of Web-based applications for the prevention of health problems by stimulating healthy behaviors.

The problem of attrition [15] poses a threat to most eHealth interventions but might pose an even bigger threat to Web-based interventions for prevention, considering that people who do not experience an urgent health problem might be less internally motivated to change their behavior [16]. Until recently, the characteristics of the users and nonusers of Web-based applications have gained only very limited attention [17-19]. It is important to know who the users of these interventions are. This knowledge helps us identify important factors in the dissemination of these interventions and the characteristics of intended users who are not reached [20]. Moreover, recent studies indicate that people react differently to motivational and persuasive strategies, which might make the need for examining user characteristics even more essential [21]. A recent review by Christensen and colleagues [22] emphasized the need for a theoretical framework to increase our understanding of attrition. They proposed using the framework adopted by the World Health Organization (WHO) [16] (ie, five dimensions of adherence: health system factors, social and economical factors, therapy-related factors, condition-related factors, and patient-related factors) and mention the possible potential of behavior theories. Furthermore, research into the reasons for use of Web-based eHealth applications can give us valuable information on what the users hope to accomplish and how the application can assist them. In addition, usability and satisfaction with an application can play an important role in the extent to which such applications are ultimately used [15,23].

We incorporated the WHO framework and behavior theories in a study of use of the Healthy Weight Assistant (HWA), a Web-based lifestyle intervention. We considered the influence of social and economic factors (demographics), condition-related factors (ie, general practitioner [GP] visits, having a chronic condition, and self-reported and self-rated dietary and PA behavior), patient-related factors or constructs identified by behavior change theories (ie, knowledge, attitude, and self-efficacy) [24-25], and reasons for use and satisfaction with the intervention.

Additionally in this study, we assessed the effectiveness of the intervention using self-reported dietary and PA behavior as primary outcome measures because the intervention was aimed at improving health behavior. We included secondary outcome measures that are known determinants of behavior change. We also chose to include measures of knowledge, attitude, and self-efficacy [24-25]. Self-rated behavior and insight into behavior were included as secondary outcome measures because one of the goals of the intervention was to improve insight into one’s own behavior.

Consequently, our research questions were: What characteristics of participants are related to the use of the HWA intervention? What effects does the HWA intervention have on the primary and secondary outcome measures?

## Methods

### Recruitment and Design

**Figure 1:**
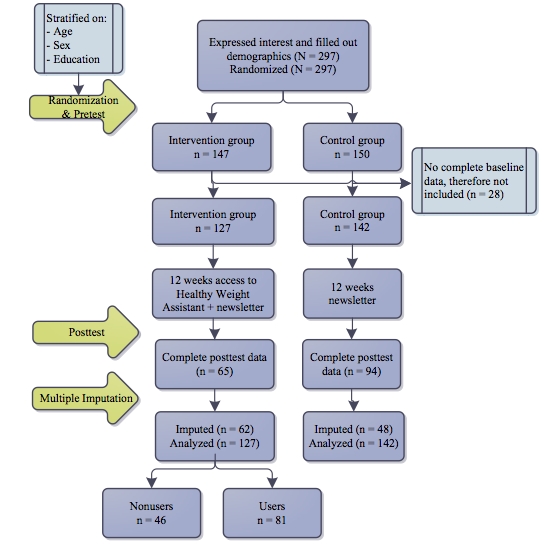
Flowchart of the study

Participants were recruited through advertisements about an online lifestyle intervention in local newspapers, supermarkets, and on health-related websites. Permission of an ethics review board for the study was not required because, according to the Dutch law, nonintrusive interventions conducted with healthy adults do not require approval from an ethics board. In total, 297 respondents expressed interest in using an online lifestyle intervention and satisfied our inclusion criteria (body mass index [BMI] 18.5 - 28.0 kg/m^2^, Dutch-speaking). The inclusion criterion for BMI was chosen to reflect the target group of the intervention under investigation. The sample used in this study was a self-selected convenience sample. Enrollment took place beginning November 1, 2008, and ending December 31, 2008. All participants were randomly assigned to either the Web-based lifestyle coach or a waiting list. A total of 150 participants were allocated to the waiting list group, and 147 participants were allocated to the intervention group. Participants filled out online questionnaires before the 12-week intervention period started and again after the intervention period ended. The posttest questionnaire was available for all respondents for a period of 3 weeks beginning February 27 and ending April 16. After the intervention period, respondents in the waiting list group could use the intervention. The flowchart of the study can be found in Figure 1.

### Randomization

Randomization took place 1 week before the start of the intervention period. We used block randomization with blocks of 4 participants, stratified on age, sex, and education. The randomization scheme was created by a computer application and carried out by a member of the research team. Participants who filled out demographic information were randomized. Only respondents who completed the pretest questionnaire were included; therefore, 28 respondents were excluded. Participants were not blinded to randomization outcome but received an email with information on when and how they were able to access the Healthy Weight Assistant (HWA) after filling out the pretest questionnaire.

### Intervention

The Healthy Weight Assistant (HWA) is a Web-based lifestyle intervention developed by the Netherlands Nutrition Centre, which is a government-funded organization focusing on increasing the knowledge of consumers about the quality of food and encouraging consumers to eat healthily and safely. The goal of the HWA is to support people with a healthy weight and people who are slightly overweight (ie, BMI 18.5-28.0 kg/m^2^) to maintain and achieve a healthy weight. The aim is not to achieve a given weight loss, but to support the achievement of healthy dietary and PA behavior. Therefore, the focus was broader than only energy balance-related behavior. The target group was selected by the Netherlands Nutrition Centre according to their BMI classification. The theoretical basis for behavior change via the HWA is the transtheoretical model [26], which entails that the participants are addressed according to the stage of change in which they find themselves when starting the application. The researchers were not the leading party in the design of the HWA but have done earlier research on the application. This previous study employed user-centered evaluation methods and has led to slight alterations in the design of the application in order to increase users’ motivation to keep using the HWA and their motivation to change behavior [27].

**Figure 2:**
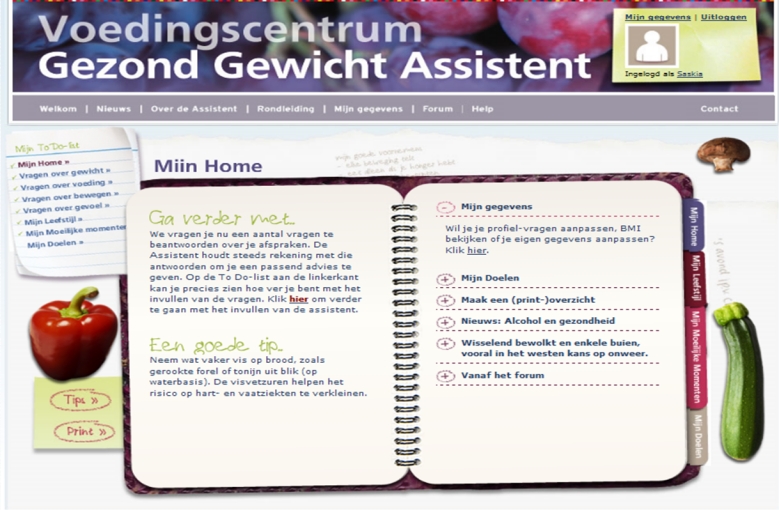
The Healthy Weight Assistant

The HWA consists of 4 steps, which are marked in the application by a “to-do list” and tabs in the “diary” (Figure 2). When users enter the program for the first time, they start by assessing their baseline status. In this step, users answer questions about their body weight, dietary behavior, physical activity behavior, and emotions concerning these behaviors. This results in tailored advice that can be applied in the next steps of the application. The second step is motivation. Users are asked about their motivation to change behavior, and the application assists them in making these motivations clear to themselves, thereby also focusing on clarifying their emotions related to behavior. The third step is called *difficult moments*. Users are encouraged to reflect on their difficult moments (ie, moments at which it is tempting to engage in unhealthy behavior) and to provide solutions for these moments. The HWA coaches the user throughout this step by giving automated tailored feedback based on input of the users. The final step is goal setting and monitoring achievement of goals. Users are coached to set useful and realistic goals and can opt to receive a weekly email reminder on these goals. Additionally, users can give feedback on the achievement of their own goals and access an overview of previous goals. The news section of the HWA is regularly updated, and when users exit the application, random hints are displayed. Other content is static. The HWA is designed to be used at regular intervals. The intended use is one or multiple visits within a short period of time to complete the first 3 steps. For the last step, the intended use is once a week to once a fortnight over a longer period of time. For the research period, the HWA was only available to the participants. After this period, the application was made openly accessible through a website.

### Waiting List

We made use of a waiting list control group. Participants randomized in this group received an email newsletter every 3 weeks, but no access to the HWA during the intervention period. The newsletter contained general information about the study and about the University of Twente. Furthermore, it contained leisure tips, but it contained no information on healthy lifestyle. After the intervention period, participants in the waiting list group received access to the HWA. Participants in the intervention group also received the newsletter every 3 weeks.

### Research Instruments 

Online questionnaires were used to assess pretest and posttest values. Education was self-reported and recoded into the following three categories: low (primary and lower vocational education), moderate (secondary and middle vocational education), and high (higher vocational and university education). BMI (kg/m^2^) was calculated using self-reported weight and length. Dietary behavior was measured using a 14-item self-report questionnaire of the Netherlands Nutrition Centre, based on the Netherlands classification model [28]. This questionnaire has not been validated but was used because of the applicability to the standards used by the Netherlands Nutrition Centre [29]. These standards are based on a report of the Health Council of the Netherlands, which is the basis of nutritional education in the Netherlands [30]. This questionnaire classifies respondents as *unhealthy* (not complying to the standards on all aspects), *improvable* (complying with the standards on some aspects), and *healthy* (complying with the standards on all aspects). This classification entails that respondents in the healthy category have limited room for improvement because they already comply with all of the standards. We have included a translation of this questionnaire in Multimedia Appendix 1. Physical activity behavior was measured according to the Dutch Standard for Healthy Physical Activity, using a validated 4-item self-report questionnaire [31]. This questionnaire classifies respondents into two categories, *unhealthy* (not complying with the standards) and *healthy* (complying with the standards). Again, this classification entails that respondents in the healthy category have limited room for improvement because they already comply with the standards. We have included a translation of this questionnaire in Multimedia Appendix 2. Self-efficacy for diet and PA were both measured using a 3-item questionnaire with a 5-point Likert scale ranging from 1 (very high) to 5 (very low) [32]. Knowledge was assessed using a 10-item true/false questionnaire based on the Netherlands classification model [28] for diet and a 10-item true/false questionnaire for physical activity based on the Dutch Standard for Healthy Physical Activity [33]. The total scores of these questionnaires range from 1 (very poor) to 10 (excellent). Attitude was measured using a 5-item questionnaire on health consciousness attitude and a 6-item questionnaire on health-oriented beliefs; all questions used a 5-point Likert-scale ranging from 1 (very unfavorable) to 5 (very favorable). These questionnaires were based on the research of Dutta-Bergman [34] and adapted to the Dutch situation. Self-rated behavior (henceforth self-rating) was assessed by 2 items, 1 on self-rated diet and 1 on self-rated PA, both using a scale from 1 (very poor) to 10 (excellent). Insight into behavior was calculated by comparing self-reported and self-rated diet and PA based on the classification used by Ronda et al [35]. Self-rating was recoded into categories to match the categories of self-reported behavior. Therefore, self-rated diet was recoded into three categories (1-4: unhealthy; 5-7: improvable; 8-10: healthy) and self-rated PA was recoded into two categories (1-5: unhealthy; 6-10: healthy). Respondents who did not meet the criteria for recommended healthy behavior but rated their own behavior as healthy were classified as overestimators. Respondents who did meet the criteria for healthy behavior but rated their behavior as unhealthy were classified as underestimators. The remaining respondents were considered to have had realistic insight into their behavior. Pretest and posttest questionnaires were identical except for the following additional items at posttest: the number of newsletters received and opened (waiting list group) and satisfaction with the HWA (intervention group). Satisfaction was measured using 4 items with a 5-point Likert scale ranging from 1 (very negative) to 5 (very positive) on user friendliness, usefulness, recommending to others, and willingness to continue using the HWA [36].

In addition to the online questionnaires, the HWA stored every log-on by a participant. These log files were used to attain the usage of the HWA, that is, the number of times each respondent logged on to the HWA within the intervention period.

### Electronic Surveys

SurveyMonkey was used for the electronic data collection [37]. The first page of the survey consisted of an informed consent. By agreeing to participate, participants were led to the actual questionnaire. Data was protected following the security measures of SurveyMonkey [38]. Moreover, no personal identifying information apart from an email address was collected. Our survey was pretested using 5 nonparticipants comparable to the participants of the study. Feedback from the pretest was implemented in the final survey.

Our format of data collection was an “open survey” [39] posted on a website. The survey was only accessible through our research website for respondents who satisfied our inclusion criteria. The initial contact mode was through online and offline advertisements for research into an online lifestyle coach. It was mandatory for participants to fill out the questionnaire to be included in the study. We offered no incentives to participate other than the use of the lifestyle coach. The pretest questionnaire was available for 8 weeks; the posttest questionnaire was available for 3 weeks. We used randomization of items for Likert-type questions with no specific order. The number of items was 42, divided over 5 screens. All questions were mandatory except comment boxes. Respondents were able to review and, if necessary, change previous answers until they had submitted the completed questionnaire.

We were not able to record unique site visitors or survey visitors. The completion rate was 90% (269/297). To prevent multiple entries from the same person we used cookies that were stored when visiting the first page and were valid for 14 days. Also, we checked IP addresses. Entries from the same address with identical sex and birth date were checked for completeness. The most complete entry was saved, or, in case of equal completeness, the first entry was saved.

### Participants

Previous research on the HWA using the same research instrument on self-reported dietary behavior yielded information on the mean and standard deviation of this primary outcome measure (mean 62.9, SD 8.43) [27]. To be able to measure a meaningful difference (3.5 points) we needed a detectable effect size of 0.4. When testing at the .05 level, and, using a power of 80%, we calculated that we needed a sample size of 200 (100 per group).

### Analyses

Statistical analyses were performed using SPSS Statistics 17.0 (IBM Corporation, Somers, NY, USA). We used the multiple imputation (MI) feature of SPSS Statistics 17.0 to handle missing data of posttest nonrespondents. Demographic variables and baseline outcome measures were used as predictors in the imputation model. We used an iterative Markov chain Monte Carlo method, which is the fully conditional specification. In addition, five imputed datasets were generated on which the effectiveness analyses were performed. When possible, pooled outcomes were used for the analyses; otherwise, the five estimates were combined into a single overall estimate following the MI inference rules of Rubin [40].

Differences between users and nonusers within the intervention group were assessed using Pearson's chi-square and analysis of variance testing. Furthermore, regression analysis was used to see whether characteristics predicted use of the intervention.

Effectiveness of the intervention was assessed by intention-to-treat (ITT) using effect sizes and odds ratios. Additionally, exploratory analyses were performed on pretest and posttest scores of all participants combined and separately for the control group, the users, and the nonusers of the intervention using regression analyses and effect sizes. All reported *P* values are 2-tailed. We used no statistical measures to correct for multiple testing. Effect sizes for differences in means are presented as Cohen’s d and effect sizes for nonparametric variables are presented as *r*, calculated from the *z* scores of the Wilcoxon signed rank test [41].

## Results

### Response Rates

Of the 269 enrolled respondents (those who completed the pretest questionnaire), 159 respondents filled out the posttest questionnaire (response rate = 59%, 159/269). The response was significantly lower in the intervention group (51%, 65/127) than in the control group (66%, 94/142) (*P* = .01). There were baseline differences between responders (ie, respondents who filled out the posttest questionnaire) and research dropouts on outcome variables. As shown in Table 1, dropouts scored significantly lower on attitude and self-rating. In addition, within the intervention group, only 48% (30/62) of dropouts used the HWA as opposed to 78% (51/65) of responders (χ^2^_1_ = 12.424, *P* < .001).

**Table 1:** Baseline differences on outcome variables between responders and dropouts

Variable		Responders (n=159)	Dropouts (n=110)	*P*
BMI (kg/m^2^), mean (SD)		24.0 (2.5)	23.9 (2.5)	.83
**Diet, n (%)**				.18
	Healthy	48 (30)	26 (24)	
	Improvable	99 (62)	69 (63)	
	Unhealthy	12 (8)	15 (14)	
Healthy PA, n (%)		64 (42)	41 (37)	.46
Knowledge, mean (SD)^a^		7.9 (1.1)	7.7 (1.2)	.19
Attitude, mean (SD)^b^		4.1 (0.4)	3.9 (0.5)	.001
Self-efficacy, mean (SD)^c^		2.1 (0.6)	2.2 (0.6)	.55
Self-rating, mean (SD)^d^		6.8 (1.1)	6.4 (1.5)	.02
Realistic insight, diet, n (%)		92 (60)	69 (63)	.35
Realistic insight, PA, n (%)		88 (58)	70 (64)	.60

^a^ Scale from 1 (very poor) to 10 (excellent)

^b^ Scale from 1 (very unfavorable) to 5 (very favorable)

^c^ Scale from 1 (very high) to 5 (very low)

^d^ Scale from 1 (very poor) to 10 (excellent)

### Descriptive Analyses of Baseline Variables 

As shown in Table 2, most of the respondents in this study were female (177/269, 66%) and in the highest education category (143/269, 53%). Mean age was 41.5 years (SD 13.5). There were no significant differences between the intervention and control group on demographic variables and reasons for use. On outcome variables, there was one significant difference at baseline, that is, respondents in the intervention group scored significantly higher on self-efficacy than respondents in the control group. Mean scores were respectively 2.2 (SD 0.6) versus 2.1 (SD 0.6) (*F*_1,267_ = 4.109, *P* = .044). The most frequently mentioned reason by respondents for wanting to use the application was to gain more insight into their own lifestyle.

**Table 2:** Baseline demographics and reasons for use

Variable		Total (N = 269)	Intervention (n = 127)	Control (n = 142)	*P*
Age (years), mean (SD)		41.5 (13.5)	41.2 (13.5)	41.7 (13.6)	.73
Sex, n female (%)		177 (66)	85 (67)	92 (65)	.80
**Education**					.71
	High, n (%)	143 (53)	69 (54)	74 (52)	
	Moderate, n (%)	87 (32)	42 (33)	45 (32)	
	Low, n (%)	39 (15)	16 (13)	23 (16)	
Chronic condition, n (%)		48 (18)	19 (15)	29 (20)	.27
**Reasons for use**^a^					
	Insight into lifestyle, n (%)	161 (60)	80 (63)	81 (57)	.38
	Living healthier, n (%)	120 (45)	61 (48)	59 (42)	.33
	Fun, n (%)	112 (42)	55 (43)	57 (40)	.62
	Lose weight, n (%)	107 (40)	56 (44)	51 (36)	.21

^a^ Multiple answers possible so cumulative percentages do not equal 100%

### Users and Nonusers

Respondents in the waiting list (control) condition reported to have opened a mean of 3.4 (SD 1.2) out of 5 newsletters. From the log files of the HWA, we know that 81 of the 127 (64%) respondents in the intervention group used the HWA at least once, while 49% (40/81) of these used the application only once. The respondent that used the HWA most frequently used it 13 times during the intervention period of 12 weeks. The median number of times HWA was used was 1.0. Of the 127 respondents in the intervention group, 4 (3%) used the application at least the intended number of times within the intervention period (ie, once a fortnight or 6 times during the 12-week period). Satisfaction with the application was assessed within the posttest questionnaire. We used only the data provided by 50 respondents who filled out the posttest questionnaire and who had accessed the HWA at least once in the intervention period. These results are depicted in Table 3. The overall mean satisfaction score for these 50 respondents was 3.0 (SD 0.74) on the 5-point scales where 1 = very negative and 5 = very positive. A score of 3.0 lies within the neutral category.

**Table 3:** Satisfaction with the Healthy Weight Assistant (n = 50)

Item	Mean (SD)	Disagree, n (%)	Neutral, n (%)	Agree, n (%)
Easy to use	3.3 (0.83)	8 (16)	22 (44)	20 (40)
Useful	2.9 (0.87)	13 (26)	25 (50)	12 (24)
Recommend to others	3.0 (0.90)	12 (24)	22 (44)	16 (32)
Keep using	2.7 (0.89)	20 (40)	22 (44)	8 (16)

Baseline differences between respondents in the intervention group who used the application (users) and the respondents in this group who did not use the HWA at least once (nonusers) are depicted in Table 4.

**Table 4:** Baseline differences between users and nonusers in the intervention group

Variable		Users (n=81)	Nonusers (n=46)	F or χ^2^	*P*
Age (years), mean (SD)		42.6 (13.2)	38.8 (13.8)	F_1,125_= 2.307	.13
Sex, n female (%)		58 (72)	27 (59)	χ^2^_1_ = 2.2	.17
**Education**				χ^2^_2_ = 0.7	.70
	High, n (%)	46 (57)	23 (50)		
	Moderate, n (%)	26 (32)	16 (35)		
	Low, n (%)	9 (11)	7 (15)		
Chronic condition, n (%)		8 (10)	11 (24)	χ^2^_1_ = 4.5	.04
BMI (kg/m^2^), mean (SD)		24.2 (2.5)	23.7 (2.3)	F_1,125_= 0.900	.35
**Diet, N (%)**				χ^2^ = 8.4	.015
	Healthy, n (%)	28 (35)	6 (13)		
	Improvable, n (%)	46 (57)	31 (67)		
	Unhealthy, n (%)	7 (9)	9 (20)		
Healthy physical activity level, n (%)		28 (37)	19 (41)	χ^2^_1_ = 0.2	.70
Knowledge, mean (SD)		7.9 (1.1)	7.4 (1.4)	F_1,125_ = 4.194	.04
Attitude, mean (SD)		4.0 (0.4)	3.9 (0.5)	F_1,125_ = 2.665	.11
Self-efficacy, mean (SD)		2.3 (0.6)	2.2 (0.6)	F_1,125_ = 0.274	.60
Self-rating, mean (SD)		6.6 (1.4)	6.5 (1.5)	F_1,125_= 0.037	.85
**Insight, diet**				χ^2^_2_ = 8.2	.02
	Underestimation, n (%)	17 (21)	2 (4)		
	Realistic insight, n (%)	52 (64)	31 (67)		
**Insight, physical activity**				χ^2^_2_ = 2.1	.36
	Underestimation, n (%)	1 (1)	1 (2)		
	Realistic insight, n (%)	47 (58)	32 (70)		

Overall, at baseline, users were healthier (scored better on dietary behavior and had a chronic condition less often) and were more knowledgeable about healthy behavior. Furthermore, users seemed to underestimate their behavior more often than nonusers, and nonusers seemed to overestimate their behavior more often than users.

To assess whether variables of the framework proposed in the introduction could be used to predict if respondents were going to use the HWA, we performed an exploratory logistic regression using the factors from the framework (social and economic, condition-related, patient-related or constructs from behavior change theories, and reasons for use). Results of this logistic regression (Table 5) showed that one variable within the social and economic factor (ie, age) and one variable within the condition-related factor (ie, chronic health condition) significantly contributed to the model. The model showed that increased age and not having a chronic condition increased the odds of having used the application (Cox & Snell *R* ^2^ = .24, Nagelkerke *R* ^2^ = .32, Model χ^2^_18_ = 34.15, *P* = .01).

**Table 5:** Logistic regression model to predict usage of the HWA

Included		Coefficient B (Standard Error [SE])	*P*	Odds Ratio (OR) (95% Confidence Interval [CI])
Constant		-12.63 (4.013)	.002	
Factor	Variable			
Social and economic	Age	0.04 (0.018)	.02	1.04 (1.00 - 1.08)
	Internet use	0.18 (0.131)	.17	1.20 (0.93 - 1.55)
	Sex	0.50 (0.504)	.32	1.65 (0.62 - 4.44)
	Education	0.13 (0.353)	.71	1.14 (0.57 - 2.28)
Condition-related	Self-rating	−0.35 (0.379)	.36	0.71 (0.34 - 1.49)
	GP visits	1.19 (0.647)	.07	3.30 (0.93 - 11.72)
	Chronic condition	2.24 (0.749)	.003	9.40 (2.17 - 40.82)
	Diet	0.71 (0.688)	.31	2.03 (0.53 - 7.80)
	PA	0.80 (0.948)	.40	2.22 (0.35 - 14.26)
	Insight, diet	0.56 (0.667)	.40	1.76 (0.48 - 6.48)
	Insight, PA	−1.00 (0.818)	.22	0.37 (0.07 - 1.83)
Patient-related	Knowledge	0.03 (0.213)	.91	1.03 (0.68 - 1.56)
	Attitude	0.57 (0.681)	.41	1.76 (0.46 - 6.69)
	Self-efficacy	0.26 (0.458)	.57	1.30 (0.53 - 3.18)
Reasons for use	Insight into lifestyle	0.47 (0.531)	.37	1.60 (0.57 - 4.55)
	Live healthier	−0.03 (0.281)	.93	0.98 (0.56 - 1.69)
	Fun	0.13 (0.165)	.44	1.14 (0.82 - 1.57)
	Lose weight	0.16 (0.122)	.18	1.18 (0.93 - 1.50)

Furthermore, we performed a linear regression to investigate whether satisfaction with the intervention HWA predicted the number of logins (Table 6). The model showed that satisfaction did not predict frequency of use (*R* ^2^ = .05, adjusted *R* ^2^ = .04).

**Table 6:** Linear regression on satisfaction predicting number of log-ins to the Healthy Weight Assistant

	B (SE)	Beta
Constant	−2.61 (1.17)	
Satisfaction	0.70 (0.38)	0.23^a^

^a^ *P* = .07

### Effectiveness

In addition, ITT analyses were performed on all outcome variables (Table 6). We found a significant but very small effect on attitude (d = 0.08) favoring the intervention group. None of the other variables showed a significant effect of the intervention.

Complementary to the ITT analyses, we performed analyses comparing the differences of the control group with the differences of the users (results not shown). These analyses did not yield any significant effects and were comparable to the results of the ITT analyses, although the effect sizes were generally larger.

**Table 7:** Intention-to-treat (ITT) analyses

Variable		Intervention (n=127)		Control (n=142)		Effect Size^a^ (ES) or OR (95% CI)
		Pretest	Posttest	Pretest	Posttest	
BMI, mean (SD)		24.0 (2.4)	24.1 (2.5)	23.9 (2.5)	24.0 (2.5)	ES: 0.07 (-0.10 – 0.24)
**Diet**						OR: 0.84 (0.44 – 1.58)
	Healthy, n (%)	34 (27)	45 (35)	40 (28)	46 (32)	
	Improvable, n (%)	77 (61)	73 (58)	91 (64)	89 (63)	
	Unhealthy, n (%)	16 (13)	9 (7)	11 (8)	7 (5)	
Healthy PA, n (%)		49 (38.6)	58 (46)	58 (41)	69 (49)	OR: 1.10 (0.60 – 2.01)
Knowledge, mean (SD)		7.7 (1.2)	7.7 (1.3)	7.9 (1.1)	7.7 (1.3)	ES: 0.15 (−0.13 to 0.42)
Attitude, mean (SD)		4.00 (0.45)	4.03 (0.45)	4.01 (0.44)	4.02 (0.45)	ES: 0.08 (0.00 – 0.16)
Self-efficacy, mean (SD)		2.2 (0.61)	2.3 (0.70)	2.1 (0.59)	2.2 (0.64)	ES: 0.04 (−0.01 to 0.17)
Self-rating, mean (SD)		6.5 (1.4)	6.9 (1.2)	6.8 (1.2)	6.9 (1.2)	ES: 0.18 (−0.04 to 0.40)
Realistic insight, diet, n (%)		83 (65)	71 (56)	83 (59)	87 (61)	OR: 0.74 (0.35 – 1.56)
Realistic insight, PA, n (%)		79 (62)	83 (65)	84 (59)	88 (62)	OR: 0.78 (0.35 – 1.74)

^a^Effect size for ratio variables presented as Cohen’s d, that is, the number of standard deviations the intervention group (I) improved more than the control group (C) (mean improvement I – mean improvement C)/pooled SD of improvement. Effect size for ordinal variables is presented as the odds ratio.

For the group as whole (independent of randomized condition), there were significant differences between pretest and posttest scores. With respect to diet (effect size *r* = −0.12), physical activity (effect size *r* = −0.09), and self-rating (effect size d = 0.21) the study seemed to have had a positive influence, although the effect was small (data not shown). These differences could not be attributed to the intervention according to the ITT analyses. As mentioned in the previous paragraph, our results showed differences between users and nonusers at baseline. Therefore, we performed exploratory analyses on these groups to investigate whether choosing to use or not to use the application led to different outcomes. Tables 8 and 9 show that we found significant differences in some groups but not in others. Contrary to what we expected, only the nonusers showed a significant improvement on diet (*r* = −0.23). Examining the data more closely revealed that the control group and, to a larger extent, the users also showed improvement, although this difference was not significant. The data showed that only the users significantly improved with respect to PA behavior (effect size *r* = −0.17). The control group showed a nonsignificant improvement, while PA behavior of the nonusers deteriorated, but the change was nonsignificant. With respect to attitude, the nonusers showed a significant improvement with a medium effect size (d = 0.28), although the absolute difference was small. With respect to self-efficacy, the control group and the nonusers showed deterioration (effect sizes respectively d = 0.14 and d = 0.33), again with small absolute differences. Lastly, the data showed that users’ self-rated behavior was more favorable at posttest than at pretest. The size of this effect was small to medium (d = 0.27). 

**Table 8:** Pretest and posttest values on outcome variables for control group, nonusers, and users

Variable		Control (n=142)		Nonusers (n=46)		Users (n=81)	
		Pretest	Posttest	Pretest	Posttest	Pretest	Posttest
BMI, mean (SD)		23.9 (2.5)	24.0 (2.5)	23.7 (2.3)	23.9 (2.5)	24.2 (2.5)	24.2 (2.5)
**Diet**							
	Healthy, n (%)	40 (28)	46 (32)	6 (13)	11 (24)	28 (35)	34 (42)
	Improvable, n (%)	91 (64)	89 (63)	31 (68)	30 (65)	46 (57)	43 (53)
	Unhealthy, n (%)	11 (8)	7 (5)	9 (20)	5 (11)	7 (9)	4 (5)
Healthy pysical activity level, n (%)		58 (41)	69 (49)	19 (41)	16 (35)	30 (37)	42 (52)
Knowledge, mean (SD)		7.9 (1.1)	7.7 (1.3)	7.4 (1.4)	7.3 (1.4)	7.9 (1.1)	7.9 (1.2)
Attitude, mean (SD)		4.0 (0.44)	4.0 (0.45)	3.9 (0.46)	4.0 (0.45)	4.0 (0.44)	4.0 (0.44)
Self-efficacy, mean (SD)		2.1 (0.59)	2.2 (0.64)	2.2 (0.62)	2.4 (0.77)	2.3 (0.61)	2.3 (0.65)
Self-rating, mean (SD)		6.8 (1.2)	6.9 (1.2)	6.5 (1.5)	6.9 (1.4)	6.6 (1.4)	6.9 (1.1)
Realistic insight, diet, n (%)		83 (59)	87 (61)	31 (67.4)	25 (54.3)	52 (64.2)	46 (56.8)
Realistic insight, PA, n (%)		84 (59)	88 (62)	32 (69.6)	27 (58.7)	47 (58.0)	56 (69.1)

**Table 9:** Effect size (ES) of the differences between pretest and posttest values on outcome variables for control group, nonusers, and users

Variable	Control (n = 142)		Nonusers (n = 46)		Users (n = 81)	
	ES^a^	*z* (*P*)^d^/ 95% CI ES^e^	ES ^a^	*z* (*P*)^d^/ 95% CI ES^e^	ES ^a^	*z* (*P*)^d^/95% CI ES^e^
BMI	0.02^b^	CI: −0.39 to 0.44	0.06^a^	CI: -0.64 – 0.77	0.03^a^	CI: -0.51 to 0.57
Diet	−0.09^c^	*z* = −1.45 (.15)	−0.23^b^	*z* = −2.22 (.03)	−0.13^b^	*z* = −1.62 (.11)
PA	−0.10^c^	*z* = −1.65 (.10)	−0.07^b^	Z = −0.71 (.48)	−0.17^b^	*z* = −2.12 (.03)
Knowledge	−0.15^b^	CI: −0.35 to 0.04	−0.08^a^	CI: −0.49 to 0.34	0.04^a^	CI: −0.20 to 0.29
Attitude	0.01^b^	CI: −0.06 to 0.08	0.28^a^	CI: 0.15 – 0.41	−0.05^a^	CI: −0.15 to 0.05
Self-efficacy	0.14^b^	CI: 0.03 – 0.24	0.33^a^	CI: 0.13 – 0.53	0.05^a^	CI: −0.09 to 0.19
Self-rating	0.15^b^	CI: −0.05 to 0.35	0.25^a^	CI: −0.18 to 0.68	0.27^a^	CI: 0.00 – 0.54
Insight, diet	−0.03^c^	*z* = −0.51 (.61)	−0.13^b^	*z* = −1.27 (.21)	−0.07^b^	*z* = −0.90 (.37)
Insight, PA	−0.01^c^	*z* = −0.49 (.62)	−0.13^b^	*z* = −1.21 (.23)	−0.11^b^	*z* = −1.42 (.16)

^a^ Effect sizes for ratio variables are presented as Cohen’s d, while effect sizes for ordinal variables are presented as *r.*

^b^ Effect size (ES) presented as Cohen’s d: (mean_post_ - mean_pre_)/SD_pooled_

^c^ Effect size presented as *r*:  *z* /√(n)

^d^ Wilcoxon signed-rank test

^e^ In this column the reliability of the effect size is presented as the confidence interval for Cohen’s d for ratio variables and as *z* statistic with *P* value for ordinal variables

## Discussion

The results showed that the HWA was not used as often as intended. Increased age and not having a chronic condition increased the odds of having used the application at least once. Moreover, users were healthier and more knowledgeable about healthy behavior than nonusers. The ITT analyses showed no apparent effects of the intervention; however, there were differences in the effect of the intervention on users and nonusers. With respect to dietary behavior and attitude, nonusers improved more than users, while with respect to physical activity and self-rated behavior the users improved more than nonusers. On self-efficacy, the control group and the nonusers showed deterioration from baseline to posttest.

Only 64% (81 out of 127) of the participants who received access to the HWA actually used the application. This finding is not unique to this study; for example, see [6,15,20,42]. This stresses an important aspect of Web-based interventions, that is, of the respondents who agree to participate in a study on a Web-based intervention, we can expect that a substantial percentage does not use the intervention at all. In addition, we saw that the HWA is not used as often as intended in the design of the application. Of the included social and economic factors of the proposed framework, only increased age increased the odds of having used the application. This finding might seem counterintuitive, but it concurs with recent findings on the motivation to use e-consultation [43], which showed that older people were more motivated to use e-consultation than younger people. With respect to the condition-related factors, the regression analysis showed that having a chronic condition decreased the odds of using the application. An explanation might be that the HWA was not developed for people with chronic conditions and no special attention is paid to the needs of people with chronic conditions. Therefore, these people might feel that the HWA does not suit their needs. Significant differences between users and nonusers on condition-related factors showed that users were healthier. A reason for this might be that people like to be rewarded for their healthy behavior and not confronted with their unhealthy behavior.

Additionally, users more often underestimated their dietary behavior (respondents who did meet the criteria for healthy behavior but who rated their behaviour as unhealthy were classified as underestimators), while nonusers more often overestimated their behavior. This shows that the people who could have benefited most from the HWA were less likely to use the application. Of the patient-related factors or constructs from behavior change theories, only knowledge showed a significant difference between users and nonusers. Users knew more about healthy behavior, which supports the notion that the people who could have benefited most from the HWA were least likely to use the application.

There were no differences related to the reasons for use between users and nonusers, and the different reasons do not explain whether respondents used the HWA or not. However, the reasons for use might play a role in the frequency of use. The most frequently mentioned reason for wanting to use the intervention was to gain insight into one’s own behavior (60%). It might be that this goal was reached after using the HWA once, and participants might not have felt the need to use the HWA again.

Interestingly, the intervention was specifically not made to help people lose weight, but this goal was mentioned by 40% of respondents. Respondents seemed to want a quick and short-term effect (to gain insight) and might not have been willing to use the intervention frequently to work on a long-term goal (eg, a healthier lifestyle). Satisfaction with the HWA was not associated with the frequency of use. However, overall, participants were not very satisfied with the HWA, which might have contributed to the relative low usage rates. To summarize, one of the social and economic factors (ie, age), condition related factors (ie, chronic condition, self-reported behavior, and insight into behavior), and one of the patient-related factors (ie, knowledge) were related to use of the system. Satisfaction and reasons for use provided more in-depth information related to the causes of the lack of adherence to the intervention.

At baseline, the intervention and control groups showed a significant difference in attitude. The absolute value of the difference was small, however, and we don’t consider it to be a meaningful difference. Therefore, we can argue that the groups were comparable at baseline. We found no meaningful significant effects of the intervention using ITT analyses. We did find that both the waiting list group and the intervention group showed significant improvement on behavior and a significantly more favorable self-rated behavior. This well-known Hawthorne effect [44] (ie, the effect on outcome through participation in research) might be explained by the increased attention participants paid to healthy behavior due to completing questionnaires on behavior and by increased awareness of current and desired behavior. Another explanation for the improvement in all respondents might be social desirability. Thinking of the intended behavior might have influenced the responses given in the posttest questionnaire. Considering the control group, the users, and the nonusers separately showed that only nonusers significantly improved on dietary behavior. This might be due to the large differences between users and nonusers at baseline. Users were already much healthier, and both groups improved, although at posttest, the nonusers were still less healthy than the users. It seems that a ceiling effect prevented the users from improving significantly while the nonusers had much room for improvement and, for that reason, showed significant improvement. On PA, we found that only the users of the intervention improved significantly, although the effect size was not very large (*r* = −0.17). The nonusers, who chose not to use the intervention, showed a decline in behavior while the control group showed improvement. Although these differences were not significant, this does point toward a difference between choosing not to use an intervention and not being able to use an intervention. However, these differences might also reflect social desirability because of the focus on PA in the intervention. Lastly, users judged their own behavior significantly more positively after the intervention period than before. None of the other groups showed this significant improvement. Summarizing, we found no apparent effects of the HWA, but it seems that having chosen to use or not to use the intervention led to different outcomes. Combined with the differences between groups at baseline, this seems to imply that these groups are truly different and should be treated as separate entities.

In this study, we were faced with substantial dropout and nonusage rates. High dropout rates are not uncommon in this field of research and have been said to be a major challenge [45,46]. Additionally, the reduction of nonusage rates is also a major challenge [15,47]. Faced with these challenges, it is important to note that in this study the groups of dropouts and nonusers overlapped, but were not the same. Almost half of the dropouts had been users, and there were also nonusers that were responders. Consequently, it is very important keep these two concepts apart.

Our results showed that the users of the HWA were healthier than nonusers, which is an unfortunate finding not unique to this study [18]. The group for which the intervention seemed to have been most useful, namely people who had room for improvement on both diet and physical activity, were less likely to have used the HWA. This tells us that we need to try different ways to entice potential users who could benefit from the HWA to become active users. More effort should be made to tempt the nonusers of the intervention to become users. One way to do this might be to make it as easy as possible to start using the application. The moment people are interested, they should be able to use the application. In our study, there was considerable time between expressing interest and being able to use the application. Moreover, participants had to check their email, click on a link, and create a profile. All these steps require effort and could thereby decrease the odds of using the intervention. Once participants become users, the application itself can stimulate adherence. This might be done by regularly providing new content, by including reminders (through email or text messaging), or by explicitly telling participants what is expected of them in terms of usage. In our view, including these aspects would have improved the HWA.

In this study, the frameworks used to predict usage and to study effectiveness seem to have been insufficient. From the WHO framework [16], some factors, especially condition-related, seem to have explanatory power but not enough to fully explain why participants choose whether to use an intervention. This might be due to the fact that the goal of the model is adherence to treatment and not adherence to technology. Moreover, attitude, self-efficacy, and knowledge do not contribute to a better understanding of the effects of the intervention. These variables from classic behavior change theories might not discriminate enough. To gain more insight into how online interventions can support people in changing their behavior, we should try to take into account the specific barriers and opportunities of eHealth interventions and integrate them into a comprehensive conceptual framework.

### Limitations

A limitation of this study is the use of self-reported behaviors. Although we used questionnaires used in previous studies, there is a chance of biased results due to social desirability or lack of insight into behavior. As a consequence, a possible change in insight into behavior might not be reflected in our results. It could be that at baseline, participants provided optimistic self-reported behavior. Due to the intervention, the users might have provided more realistic self-reported behavior at posttest. Unfortunately, this potentially positive effect of the HWA could not be tested in this study. A second limitation is related to the participants in this study. Most respondents were female and highly educated. Various studies have reported overrepresentation of this group [6,18,48,49]. Nevertheless, the question remains whether these results can be generalized to the broader target population of the HWA. Another limitation of this study is that we measured the usage of the system as the number of log-ons per participant. What participants did while logged on and for how long they were logged on, we do not know. As more and more eHealth research takes the usage of the applications into account, it might be beneficial to standardize the assessment of usage. Furthermore, a limitation of this study is related to the response rate. Our overall response rate was quite low (59%), and we found significant differences between responders and nonresponders. We accounted for this bias by using multiple imputation procedures. However, imputing 41% of the data might have yielded unreliable estimates, although research has shown that imputing up to 58% can be more reliable than listwise deletion [40,50]. In our view, this study has provided valuable insights into the users of a Web-based intervention. However, had we been able to conduct this study again, we would have changed the way we dealt with several issues. First of all, we would have included a larger number of respondents to certify a sample size large enough to account for the high dropout and nonusage rates. Second, we would have tested and adapted the application during development so that we could have chosen the outcome measures and study period to better reflect the goals and expected effects of the application. Unfortunately, this was not possible in the current study, and this stresses the importance of a close collaboration between researchers and developers of eHealth interventions.

### Future Work

Usage is a major issue in research into the effects of eHealth applications. More research is needed into transforming potential users into actual users and into keeping them engaged with the application and, thereby, stimulating them to keep using the intervention. Moreover, long-term research on the use of eHealth applications is needed to provide insight into the way usage fluctuates over time. From this study, we have gained insight into differences between users and nonusers, which can be seen as a first step to decreasing attrition. The next step might be found when looking at the opportunities technology has to offer. For example, several recent studies have shown beneficial effects of adding mobile technology [51-53] and devices that provide automated tailored feedback [54]. Additionally, the field of persuasive technology might provide us with insight into how technology as a medium can persuade and motivate users to change behavior [55,56].

## Multimedia Appendix 1

Translated questionnaire dietary behavior

## Multimedia Appendix 2

Translated questionnaire physical activity behavior

## Abbreviations

BMI: body mass index
CI: confidence interval
ES: effect size
GP: general practitioner
HWA: Healthy Weight Assistant
ITT: intention-to-treat
OR: odds ratio
PA: physical activity
SE: standard error
WHO: World Health Organization
