# Supplementary material for: Effectiveness of a Web-based Intervention Aimed at Healthy Dietary and Physical Activity Behavior: A Randomized Controlled Trial About Users and Usage
Source: J Med Internet Res. 2011 Apr 14;13(2):e32. doi: 10.2196/jmir.1624 (PMC3221377; doi:10.2196/jmir.1624)
Supplement: Supplementary file 1 [file jmir_v13i2e32_app1.pdf]

Translated Questionnaire Dietary Behavior

Werkman A, Hammink A, Netherlands Nutrition Centre. Vragenlijst & Scoreberekening voor gezonde versus ongezonde voeding. 2008

Translated from Dutch

1. How many times in the past four weeks have you eaten vegetables?
Never
Almost never
Less than once a week
1 or 2 times a week
3 or 4 times a week
5 or 6 times a week
Every day

2. How many times in the past four weeks have you eaten fruits?
Never
Almost never
Less than once a week
1 or 2 times a week
3 or 4 times a week
5 or 6 times a week
Every day

3. How many times in the past four weeks have you drunk/eaten milk and/or milk products?
Never => go to question 5
Almost never => go to question 5
Less than once a week
1 or 2 times a week
3 or 4 times a week
5 or 6 times a week
Every day

4. What kind of milk and/or milk products did you usually drink/eat?
Skimmed milk and/or milk products, like skimmed milk, skimmed yoghurt, buttermilk and/or skimmed custard
Low-fat milk and/or milk products, like low-fat milk, low-fat yoghurt and/or low-fat custard
Whole milk and/or milk products, like whole milk, whole yoghurt and/or whole custard
Varying low-fat and skimmed
Varying low-fat and whole
Varying skimmed, low-fat and whole

5. How many times in the past four weeks have you eaten bread?

Never => go to question 8
Almost never => go to question 8
Less than once a week
1 or 2 times a week
3 or 4 times a week
5 or 6 times a week
Every day

6. What kind of bread did you usually eat?
Brown, whole wheat and/or rye bread
White bread
Varying

7. What kind of butter did you usually use on your bread?
(low-fat) margarine
Butter
Varying
I do not use butter on bread

8. How many times in the past four weeks have you used fat or (olive)oil when preparing a meal?
Never => go to question 10
Almost never => go to question 10
Less than once a week
1 or 2 times a week
3 or 4 times a week
5 or 6 times a week
Every day

9. What kind of fat did you usually use?
Margarine from a tub and/or squeeze-bottle, (olive)oil and/or liquid frying fat
A packet of margarine and/or solid baking, frying or deep frying fat
Butter

10. How many times in the past four weeks have you eaten meat, fish or chicken with your hot meal?
Never => go to question 12
Almost never => go to question 12
Less than once a week
1 or 2 times a week
3 or 4 times a week
5 or 6 times a week
Every day

11. What kind of meat, fish or chicken did you usually eat with your hot meal?

Lean kinds (like steak tartare, pork tenderloin, (pork) steak, chicken breast, roast beef, ham, loin chop, tilapia, pike perch and the like)
Fatter kinds (like minced meat, hamburger, sausage, shoulder chop, bacon, mackerel, salmon and the like)
Varying

12. How many times in the past four weeks have you drunk regular soda, sports drinks and/or juices (not LIGHT soda)?
Never
Almost never
Less than once a week
1 or 2 times a week
3 or 4 times a week
5 or 6 times a week
Every day

13. How many times in the past four weeks have you consumed alcoholic beverages?
Never
Almost never
Less than once a week
1 or 2 times a week
3 or 4 times a week
5 or 6 times a week
Every day

14. How many times in the past four weeks have you eaten large biscuits, cake, candy bars, snacks, candy and/or crisps in between meals?
Never
Almost never
Less than once a week
1 or 2 times a week
3 or 4 times a week
5 or 6 times a week
Every day

This is a Multimedia Appendix to a full manuscript published in the J Med Internet Res, for full copyright and citation information see http://dx.doi.org/10.2196/jmir.1624
