# Supplementary material for: Effectiveness of a Web-based Intervention Aimed at Healthy Dietary and Physical Activity Behavior: A Randomized Controlled Trial About Users and Usage
Source: J Med Internet Res. 2011 Apr 14;13(2):e32. doi: 10.2196/jmir.1624 (PMC3221377; doi:10.2196/jmir.1624)
Supplement: Supplementary file 2 [file jmir_v13i2e32_app2.pdf]

Translated Questionnaire Physical Activity Behavior

Douwes M, Hildebrandt VH. Vragen naar de mate van lichamelijke activiteit. Geneeskd Sport 2000;33(1):9-16.

Translated from Dutch:

The following questions are about physical activity, such as walking, biking, gardening, sporting or moving at work or at school.

1. How many days a week have you performed this kind of physical activity for at least 30 minutes a day during SUMMER? It is about the average number of days in a regular week.
Less than 1 day a week
1 day a week
2 days a week
3 days a week
4 days a week
5 days a week
6 days a week
7 days a week

2. How many days a week have you performed this kind of physical activity for at least 30 minutes a day during WINTER? It is about the average number of days in a regular week.
Less than 1 day a week
1 day a week
2 days a week
3 days a week
4 days a week
5 days a week
6 days a week
7 days a week

The following questions are about strenuous activities in your free time.

3. In your free time during summer, how many times do you carry out strenuous sports or physical activities that take long enough to become sweaty? It is about strenuous physical activity that lasts at least 20 minutes each time.
Less than 1 day a month
1 day a month
2 days a month
3 days a month
4 days a month
2 days a week
3 days a week
4 days a week
5 days a week
6 days a week
7 days a week

3. In your free time during winter, how many times do you carry out strenuous sports or physical activities that take long enough to become sweaty? It is about strenuous physical activity that lasts at least 20 minutes each time.
Less than 1 day a month
1 day a month
2 days a month
3 days a month
4 days a month
2 days a week
3 days a week
4 days a week
5 days a week
6 days a week
7 days a week

This is a Multimedia Appendix to a full manuscript published in the J Med Internet Res, for full copyright and citation information see http://dx.doi.org/10.2196/jmir.1624
